# Hierarchical Representation of Multistep Tasks in Multiple-Demand and Default Mode Networks

**DOI:** 10.1523/JNEUROSCI.0594-20.2020

**Published:** 2020-09-30

**Authors:** Tanya Wen, John Duncan, Daniel J Mitchell

**Affiliations:** ^1^Medical Research Council, Cognition and Brain Sciences Unit, University of Cambridge, Cambridge CB2 7EF, United Kingdom; ^2^Department of Experimental Psychology, University of Oxford, Radcliffe Observatory, Oxford OX2 6GG, United Kingdom

**Keywords:** default mode network, fMRI, hierarchy, multiple-demand network, representational similarity analysis, task episodes

## Abstract

Task episodes consist of sequences of steps that are performed to achieve a goal. We used fMRI to examine neural representation of task identity, component items, and sequential position, focusing on two major cortical systems—the multiple-demand (MD) and default mode networks (DMN). Human participants (20 males, 22 females) learned six tasks each consisting of four steps. Inside the scanner, participants were cued which task to perform and then sequentially identified the target item of each step in the correct order. Univariate time course analyses indicated that intra-episode progress was tracked by a tonically increasing global response, plus an increasing phasic step response specific to MD regions. Inter-episode boundaries evoked a widespread response at episode onset, plus a marked offset response specific to DMN regions. Representational similarity analysis (RSA) was used to examine representation of task identity and component steps. Both networks represented the content and position of individual steps, however the DMN preferentially represented task identity while the MD network preferentially represented step-level information. Thus, although both MD and DMN networks are sensitive to step-level and episode-level information in the context of hierarchical task performance, they exhibit dissociable profiles in terms of both temporal dynamics and representational content. The results suggest collaboration of multiple brain regions in control of multistep behavior, with MD regions particularly involved in processing the detail of individual steps, and DMN adding representation of broad task context.

**SIGNIFICANCE STATEMENT** Achieving one's goals requires knowing what to do and when. Tasks are typically hierarchical, with smaller steps nested within overarching goals. For effective, flexible behavior, the brain must represent both levels. We contrast response time courses and information content of two major cortical systems—the multiple-demand (MD) and default mode networks (DMN)—during multistep task episodes. Both networks are sensitive to step-level and episode-level information, but with dissociable profiles. Intra-episode progress is tracked by tonically increasing global responses, plus MD-specific increasing phasic step responses. Inter-episode boundaries evoke widespread responses at episode onset, plus DMN-specific offset responses. Both networks represent content and position of individual steps; however, the DMN and MD networks favor task identity and step-level information, respectively.

## Introduction

Purposeful behavior requires retrieval of memorized sequences (Hsieh and Ranganath, 2015) to guide current actions, with overarching goals or “task episodes” (e.g., “make stew”) decomposed into achievable steps (“wash vegetables” … “chop” … “cook”; Cooper and Shallice, 2000; Schneider and Logan, 2006; Duncan, 2010). As each step is completed, its specific content loses relevance, while higher-level representations of the full task remain in behavioral control. This raises the question of how brain regions cooperate to execute a current step while keeping an overall goal in mind.

Previous literature highlights the importance of a frontoparietal multiple-demand (MD) network in controlling complex mental programs (Dosenbach et al., 2006; Duncan, 2010, 2013). MD regions are recruited during many cognitively demanding tasks (Duncan and Owen, 2000), are sensitive to hierarchical task structure (Farooqui et al., 2012; Desrochers et al., 2015; Badre and Nee, 2018), and necessary for effective problem-solving (Woolgar et al., 2010). They preferentially represent task-relevant information (Asaad et al., 2000; Everling et al., 2002; Li et al., 2007), and radically change activity patterns across successive task steps (Sigala et al., 2008).

The “default mode” network (DMN; Raichle et al., 2001) is often anti-correlated with MD activity (Fox et al., 2005b). Many findings suggest a role in attention to internal representations, uncoupled from external stimuli (Golland et al., 2006; Buckner et al., 2008), including when memories guide behavior (Konishi et al., 2015; Murphy et al., 2019) and especially when sematic associations are available (Murphy et al., 2018). Thus, DMN involvement is also expected when behavior requires recall of learned task sequences.

Steps within a task plan resemble events within episodic memory (Ezzyat and Davachi, 2011). Humans are proposed to segment episodes into temporally meaningful chunks, separated by event boundaries (Zacks and Tversky, 2001; Radvansky and Zacks, 2017). Event boundaries may activate MD-like regions (Zacks et al., 2001; Sridharan et al., 2007), but also areas associated with episodic memory, including hippocampus (Ben-Yakov et al., 2013; Ben-Yakov and Henson, 2018) and DMN (Speer et al., 2007), or both (Ezzyat and Davachi, 2011). The DMN is implicated in high-level cognition at a temporally and conceptually broad scale, including representation of schemas (Robin and Moscovitch, 2017), situation models (Reagh and Ranganath, 2018), and task-sets (Crittenden et al., 2015), and responds especially to boundaries rated as separating long, meaningful events (Speer et al., 2007). Temporal scrambling of narrative stimuli suggests a cortical hierarchy of temporal receptive windows (Lerner et al., 2011), with short-timescale processing in sensory regions, through intermediate timescales in MD regions, to longest-timescale processing in DMN regions (Chen et al., 2016), consistent with a gradient from sensorimotor to transmodal cortex (Margulies et al., 2016). Investigation of multivoxel pattern transitions during narrative perception (Baldassano et al., 2018) found the longest-timescale event representations in posterior medial cortex and the intraparietal sulcus (IPS), within the DMN and MD network, respectively, while neural event structure was abstracted from sensory modality around the temporoparietal junction and in lateral frontal cortex (LFC), again within DMN and MD networks, respectively. Overall, the literature suggests that both DMN and MD networks are potentially well-suited to representing temporally extended task episodes.

The distinct roles of the DMN and MD networks in representing different aspects of task episodes remain unclear. We therefore examined how these networks represent information at multiple levels of abstraction within a task: individual steps, including content and position within an episode, whole tasks, and groups of related tasks. Participants learned four-step tasks associated with different rooms, and then sequentially identified target items corresponding to each step of cued tasks. Thus, we could quantify neural representation of rooms (e.g., kitchen), tasks (e.g., “make stew”), step position (e.g., third) and items associated with steps (e.g., “wash vegetables”). We used univariate analyses to characterize temporally evolving activity across episodes, and representational similarity analysis (RSA) to investigate representations of task structure and content. We hypothesized that the MD and DMN networks would be preferentially sensitive to different levels of the temporal task hierarchy.

## Materials and Methods

### Participants

42 participants (20 males, 22 females; ages 18–39, mean = 26.79, SD = 4.77) were included in the experiment at the MRC Cognition and Brain Sciences Unit. An additional 19 participants were excluded [two were discovered to have cysts, one lost several slices because of poor bounding box positioning, 10 were excluded because of having no correct episodes for at least one combination of cued task × distractor task (see later), and a further six were excluded because of excessive head motion >5 mm]. Of the 10 participants that were excluded because of insufficient correct episodes, most self-reported that they had trouble concentrating and were falling asleep in the scanner, and displayed lapses in responding. This may have been a consequence of using relatively long blocks (∼28 min). All participants were neurologically healthy, right-handed, with normal or corrected-to-normal vision. Procedures were conducted in accordance with ethical approval obtained from the Cambridge Psychology Research Ethics Committee, and participants provided written, informed consent before the start of the experiment.

### Stimuli and task procedures

**Figure 1.**
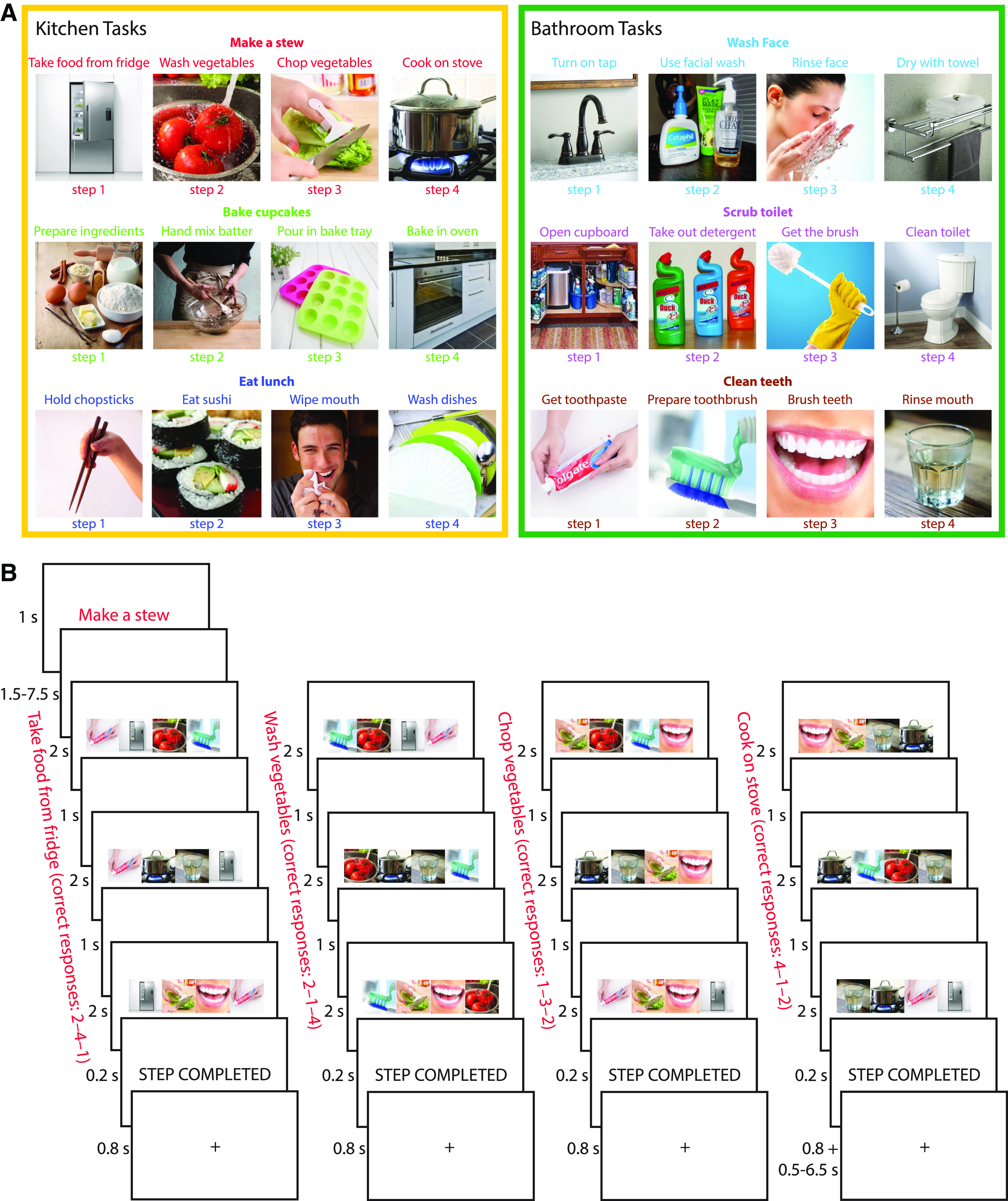
***A***, Illustration of the six task episodes (three kitchen and three bathroom tasks) memorized before going into the scanner. Each task consisted of four steps to be completed in serial order (e.g., the task “make a stew” consisted of “take food from fridge,” “wash vegetables,” “chop vegetables,” “cook on stove”). ***B***, Structure of an example task episode. Episodes began with a cue indicating which task to perform (e.g., “make a stew”). After a short delay, the first search array of four items appeared, and participants were asked to select the item corresponding to the first step of that task (here, “take food from fridge”). Participants selected this same target in three search arrays (total step duration = 9 s), then were given a brief indicator that the step had been completed, and moved on to the next step (here “wash vegetables”). Completion of all four steps completed the entire task episode.

The study consisted of a learning session outside the scanner and an execution session in the scanner. During the learning session, participants learned six everyday task sequences, each based in one of two locations (“rooms”; three kitchen and three bathroom). Each task consisted of four ordered “steps.” For example, the task “make a stew” consisted of the steps “take food from fridge,” “wash vegetables,” “chop vegetables,” “cook on stove.” Each step was associated with a unique image (“item”). The complete set of stimuli is shown in Figure 1*A*.

In the learning session, participants viewed the names and images of the steps of each task episode in sequential order. The step images were presented simultaneously with a background image corresponding to the room in which they occur (kitchen or bathroom). The learning was self-paced, in separate runs for each room. Within each room, each task sequence was presented three times, and each item within the sequence was presented until the participant decided to move on to the next item. There was a 1.5 s interstimulus interval between items. After viewing all six sequences, participants were tested for their memory of the task episodes by (1) sorting picture cards representing all steps of the six task episodes into the correct sequences, and (2) completing a pen-and-paper test in which they were asked to write down the names of the steps in the correct order for each task episode. Most participants performed both tests without error. A few participants made a mistake on one to two items but were able to correct their answers after this was pointed out. The tests ensured participants had memorized the specific step sequence of each task. Before entering the scanner, participants practiced a shortened version of the main experiment, containing one episode of each task. During scanning, participants performed two runs of the experiment, interleaved with shorter runs (∼5 min) of a localizer task that was not analyzed and is not described further.

Figure 1*B* illustrates the structure of the task episodes paradigm. At the start of each 45 s episode, participants were presented with a cue (e.g., “make a stew”) for 1 s, indicating which task to complete. This was followed by a fixation period lasting between 1.5 and 7.5 s, selected randomly from a uniform distribution, before the onset of the first step. On each step, participants had to perform three visual searches. On each search, an array of four images was presented in a horizontal row (total left to right visual angle ∼12.6°). These included (randomly ordered from left to right): (1) the correct image (“target”) corresponding to the current task step; (2) a distractor image representing a random incorrect step from the correct task; (3) a distractor representing the correct step but from an incorrect task (“distractor task”); and (4) an additional distractor representing the same incorrect step as (2), from the same incorrect task as (3). To ensure that each display contained two images from each room, distractor tasks were selected at random from the alternative room to the cued task. The array remained for 2 s, and within this time, the participant had to indicate the position of the target image using a 4-choice button box with their right hand. A 1 s fixation interval preceded onset of the next search array. Each step thus lasted for 9 s, with the participant selecting the same target in each of three search events, to allow separation of the hemodynamic response to successive task steps, while ensuring sustained focus on the relevant item within each step. At the end of the third search event, a 0.2 s presentation of the words “STEP COMPLETED” indicated the completion of that step, followed by a 0.8 s fixation interval. Without further cueing, the participant then moved on to the next task step. After completing the last step, a fixation interval of 0.5–6.5 s was presented before the onset of the cue for the next task. The total interval between the last step of the previous task and the first step of the next task was fixed at 9 s. Participants were not given feedback on their accuracy. Each run consisted of 36 task episodes (with an additional dummy episode to start), constructed so that each task appeared following each possible preceding task once. Task ordering was chosen before the start of each run to maximize the design efficiency (Dale, 1999) of all pairwise contrasts between tasks. A total of 1000 task orders were simulated, and the most efficient one was chosen. Each of the two runs lasted ∼28 min.

### fMRI data acquisition and preprocessing

Scanning took place in a 3T Siemens Prisma scanner. Functional images were acquired using a multiband gradient-echo echoplanar imaging (EPI) pulse sequence (TR = 1373 ms, TE = 33.4 ms, flip angle = 74°, 96 × 96 matrices, slice thickness = 2 mm, no gap, voxel size 2 × 2 × 2 mm, 72 axial slices covering the entire brain, four slices acquired at once). The first five volumes served as dummy scans and were discarded to avoid T1 equilibrium effects. Field maps were collected at the end of the experiment (TR = 400 ms, TE = 5.19 ms/7.65 ms, flip angle = 60°, 64 × 64 matrices, slice thickness = 3 mm, 25% gap, resolution 3 mm isotropic, 32 axial slices). High-resolution anatomical T1-weighted images were acquired for each participant using a 3D MPRAGE sequence (192 axial slices, TR = 2250 ms, TI = 900 ms, TE = 2.99 ms, flip angle = 9°, field of view = 256 × 240 × 160 mm, matrix dimensions = 256 × 240 × 160, 1-mm isotropic resolution).

The data were preprocessed and analyzed using automatic analysis (aa) pipelines and modules (Cusack et al., 2014), which called relevant functions from Statistical Parametric Mapping software (SPM 12; http://www.fil.ion.ucl.ac.uk/spm) implemented in MATLAB (The MathWorks). EPI images were realigned to correct for head motion using rigid-body transformation, unwarped based on the field maps to correct for voxel displacement because of magnetic-field inhomogeneity, and slice time corrected. The T1 image was coregistered to the mean EPI, and then coregistered and normalized to the MNI template. The normalization parameters of the T1 image were applied to all functional volumes. The data and models (see below) were temporally high-pass filtered with a cutoff at 1/128 Hz. Spatial smoothing of 10-mm full width at half maximum (FWHM) was applied for the univariate whole-brain analysis, but not for the univariate region of interest (ROI) analysis or before multivariate analysis.

### ROIs

For the primary analysis, we focused on the MD and DMN networks (Fig. 4). The MD network was based on data from Fedorenko et al. (2013, their Fig. 2), selecting frontoparietal regions responsive to cognitive demands across seven diverse tasks (http://imaging.mrc-cbu.cam.ac.uk/imaging/MDsystem). The DMN network was taken from Yeo et al. (2011), combining three subnetworks from the 17 network parcellation (numbers 15, 16, and 17; Andrews-Hanna, 2012). The left and right hemispheres were averaged and projected back to both hemispheres to create a symmetrical volume (similar to Fedorenko et al., 2013). The combined networks were then smoothed at 4-mm FWHM to eliminate isolated voxels.

**Figure 2.**
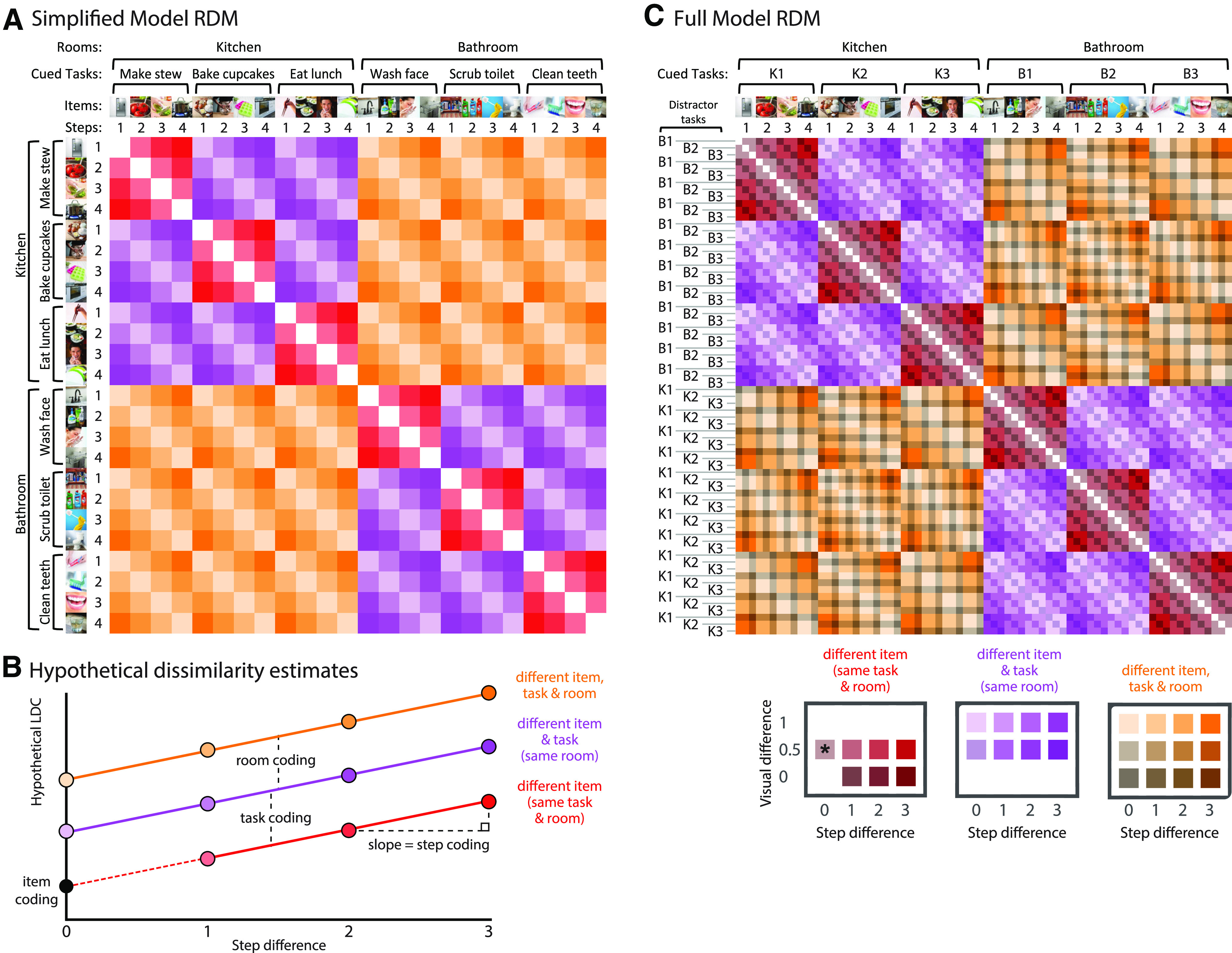
Illustration of RSA. ***A***, Simplified conceptual RDM. LDC dissimilarities are computed between every possible pair of events (six cued tasks × four steps), generating a 24 × 24 RDM. Diagonal cells are zero by definition as they reflect dissimilarity between identical events. Off-diagonal cells reflect pattern dissimilarity between events that always differ in search item, with varying additional differences in room, cued task, and step. These included event pairs that shared the same cued task (red cells), shared the same room but different cued task (purple cells), or differed in both room and cued task (orange cells). Saturation indicates the difference in steps between event pairs. ***B***, Hypothetical pattern dissimilarities resulting from room, task, and item representation across step differences. Item representation can be estimated as the intercept, i.e., estimated LDC dissimilarity in in the absence of room, task, or step differences. ***C***, Full model RDM. LDC dissimilarities were computed between every possible pair of event types (six cued tasks × four steps × three distractor tasks), generating a 72 × 72 RDM. Colors as in ***A***. Saturation indicates the difference in steps between event pairs, and brightness indicates the difference in the possible stimuli presented in the visual search arrays (see main text). The six tasks are labeled K1, K2, K3, B1, B2, B3 (with K indicating a kitchen task and B indicating a bathroom task). The asterisk indicates the only case where item is matched; this was excluded from the model fit, so that the intercept could be interpreted as item representation.

Both the MD network (Dosenbach et al., 2006, 2007; Crittenden et al., 2016) and the DMN (Andrews-Hanna et al., 2010; Andrews-Hanna, 2012; Wen et al., 2020) can be divided into finer components or subsystems, and following whole-network analysis, we examined separate subregions within each network. MD component ROIs were separated as described in Mitchell et al. (2016), based on proximity to local maxima in the data of Fedorenko et al. (2013, their Fig. 2); they included three clusters along the anterior, middle, and posterior middle frontal gyrus (aMFG, mMFG, and pMFG), a posterior-dorsal region of LFC (pdLFC) in the superior precentral sulcus, and clusters in the IPS, anterior insula (AI), and anterior cingulate cortex (ACC). DMN component ROIs were defined as spatially separate clusters within the overall network, consisting of the medial prefrontal cortex (MPFC) and posterior cingulate cortex (PCC) along the midline, as well as the inferior frontal gyrus (IFG), inferior parietal lobule (IPL), parahippocampal cortex (PHC), and parts of the lateral temporal cortex extending to the temporal pole (Temp). Overlapping voxels of the AI and IFG were excluded from each ROI and their corresponding networks. Analyses were first performed using each network as a single large ROI, and then within each component ROI to examine more fine scale differences within each network. We controlled the false discovery rate (FDR) to correct for multiple comparisons across the number of networks (2) and component ROIs (13), respectively (Benjamini and Yekutieli, 2001).

### Univariate analysis

#### Finite impulse response (FIR) model

Statistical analyses were performed first at the individual level, using a general linear model (GLM). To capture the BOLD time course throughout each task episode, as well as transitions between episodes, we modeled each consecutive pair of episodes. The first (dummy) episode of each run was separately modeled and not analyzed. For the remaining data, a 90 s period starting from the onset of the first search array of every even number episode to the first search array of the next even number episode was modeled using an FIR basis set of 60 1.5 s boxcar regressors. In this way, the response throughout task episodes could be modeled without making assumptions about the shape of the hemodynamic response. Episodes with a high proportion of errors (episodes that had >25% errors) were defined as error episodes, with the total number of error episodes per participant ranging from 0 to 6 (mean = 0.95, SD = 1.43). Episode pairs that contained at least one error episode were removed from the analysis using a similar but separate set of regressors. Effects of cues, and errors on individual search arrays, were also modeled separately, by convolving the duration of their respective events (1 s for cues and 2 s for error events) with a canonical hemodynamic response function. The six motion parameters and block means were included as regressors of no interest. Across the 90 s period, estimates for each FIR time bin were extracted from each whole network or component ROI, averaged over voxels within the region and across the six tasks. These average β estimates for individual participants were entered into a random effects group analysis.

##### Event-based GLM analysis

To complement the FIR model, an event-based GLM analysis was performed. The 9 s duration of each step allows for some separation of substep response dynamics, despite the sluggishness of the BOLD response. Previous work has separated increasing from decreasing responses on a similar timescale (Krueger et al., 2017); here we separate brief, phasic activity linked to the onset of each step, from sustained, tonic activity across the whole duration of each step. To control for the degree of visual difference between the search arrays of pairs of episodes, each combination of cued task × distractor task was modeled separately. For each combination, each step was modeled using two regressors, an onset regressor modeled with 0 s duration and an epoch regressor modeled with 9 s duration. Additionally, an offset regressor modeled with 0 s duration was placed at the end of the episode. Thus, the first onset regressor and the final offset regressor captured transient responses to episode boundaries, while the regressors modeling the onset of steps 2–4 captured phasic responses to transitions between steps within an episode; epoch regressors captured more sustained responses associated with each step. Each regressor was convolved with the canonical hemodynamic response function. There were accordingly 162 regressors of interest, two (onset and epoch) for each of the four steps and one for the offset of the entire episode in each combination of six tasks × three possible distractor tasks from the other room (for example, the target task “make a stew” could be paired with distractor tasks “wash face,” “scrub toilet,” or “clean teeth”). The maximum absolute correlation between any pair of regressors was 0.5. Error episodes (defined as episodes that had >25% errors) were removed from the analysis using a similar but separate set of regressors. The cue was modeled separately using a similar combination of onset (0 s duration) and epoch (duration from cue onset to the onset of the first task step) regressors. Motion parameters and block mean regressors were included as before. Beta estimates were averaged across the 18 cued task × distractor task combinations for individual participants, and entered into random effects group analyses. We first examined the mean effect of onset/offset and epoch regressors versus implicit baseline (with FDR correction across ROIs). Repeated measures ANOVAs were then used to examine changes across steps, including linear and quadratic trends. To complement the ROI analyses, contrasts were also conducted at the whole-brain level, using a voxel-wise FDR-corrected threshold of *p* < 0.025 per tail. Results were rendered using MRIcroGL (www.nitrc.org/projects/mricrogl).

### RSA analysis

We performed RSA using the linear discriminant contrast (LDC) to quantify dissimilarities between activation patterns. The analysis used the RSA toolbox (Nili et al., 2014), in conjunction with in-house software. The LDC was chosen because it is multivariate noise-normalized, potentially increasing sensitivity, and is a cross-validated measure which is distributed around zero when the true distance is zero (Walther et al., 2016). The LDC also allows inference on contrasts of dissimilarities across multiple pairs of task events. A pattern for each step of each combination of cued task and distractor task was obtained, by averaging the onset and epoch responses from the event-based GLM described above. This resulted in 72 patterns in total in each run. For each pair of patterns, the patterns from run 1 were projected onto a Fisher discriminant fitted for run 2, with the difference between the projected patterns providing a cross-validated estimate of a squared Mahalanobis distance. This was repeated projecting run 2 onto run 1, and we took the average as the dissimilarity measure between the two patterns. All pairs of pattern dissimilarities therefore formed a symmetrical representational dissimilarity matrix (RDM) with zeros on the diagonal by definition. To compare dissimilarity magnitude across ROIs of different sizes, the LDC values were normalized by dividing by the number of voxels within each ROI.

### Representation of information within ROIs

As for univariate analyses, we first performed RSA analysis using activation patterns from the DMN and MD networks treated as single large ROIs, and then repeated it on component ROIs. To introduce measures for room, task, step, and item representation, Figure 2*A* shows a simplified version of the full 72 × 72 RDM, collapsing across distractor task to produce just a 24 × 24 matrix. In this matrix, each cell represents a cross-validated LDC dissimilarity between the corresponding two task events. These included event pairs that shared the same cued task (red cells; e.g., “take food from fridge” and “wash vegetables”); events that shared the same room but different cued tasks (purple cells; e.g., “take food from fridge” and “hand mix batter”); and events that differed in both cued task and room (orange cells; e.g., “take food from fridge” and “use facial wash”). All event pairs additionally differed in item. Saturation of the colors is used to indicate the difference in steps between event pairs. The cells on the diagonal (white) are zero by definition as they do not reflect a comparison between different task events.

To extract measures for room, task, step and item representation, we fit values in the matrix with the regression model illustrated in Figure 2*B*. In this model, the LDC estimate for any entry in the matrix is the linear sum of components from differences in target item (contributing equally to all cells; major diagonal of the matrix ignored), room, task, and step. As a measure of step representation, we used the slope of the function relating LDC estimate to step difference. For example, steps 1 and 2 have a step difference of 1, while steps 1 and four have a step difference of 3. As a measure of room representation, we used the difference in LDC estimate for different room and same room/different task cases (Fig. 2*B*, orange vs purple). As a measure of task representation, we used the difference in LDC dissimilarity for same room/different task and same task cases (Fig. 2*B*, purple vs red). As item difference contributed similarly to all cells, it was estimated as the intercept of the full model (Fig. 2*B*, black dot).

For the actual fitting, we used a more complex model based on the full 72 × 72 RDM, used to remove a potential visual confound (Fig. 2*C*). For any cell in the full RDM, search arrays could share items from zero, one, or two tasks. For example, consider an episode with cued task “make a stew” and distractor items coming from the distractor task “wash face.” Search arrays from this episode would share no items with search arrays from an episode of “bake cupcakes” with distractors from “scrub toilet”; arrays would share items from one task when compared with the episode “make a stew” with distractors from “wash face”; arrays would share items from both tasks when compared with the episode “wash face” with distractors from “make a stew.” In the full model, we added an additional regressor to remove this potential visual confound. This was defined as “visual difference,” with values of 1 for no shared tasks, 0.5 for one shared task, and 0 for two shared tasks. The mean model coefficients across subjects were tested against zero using 1-tailed *t* tests, and multiple comparisons across ROIs were corrected using FDR < 0.05 per measure.

To account for the possibility that differences in reaction time (RT) between conditions might contribute to the neural pattern differences, a subsequent control analysis added RT difference as an extra covariate in the model. For each participant, the matrix of signed RT differences between all pairs of task steps from one run were multiplied element-wise by the signed differences from the other run. This resulted in an RDM containing a cross-validated measure of RT differences per participant, calculated in a way analogous to the brain-derived LDC RDM, again with an expected value of zero if there is no true RT difference. The regression model for LDC values was then re-calculated, covarying these cross-validated RT differences.

### Searchlight analyses

To test for representation of task information outside the predefined networks, we implemented a whole-brain searchlight procedure (Kriegeskorte et al., 2006) to perform pattern analyses in spherical ROIs (radius = 10 mm) centered on every voxel of the brain in turn. The procedure was identical to that described in the ROI analysis. Pairwise dissimilarities were derived from the 72 × 72 RDM in each sphere, and modeled as a linear combination of differences in room, cued task, step and visual search array items. Model coefficients were assigned to the central voxel of each sphere, resulting in whole-brain maps of information representation for each participant. These maps were smoothed with a 10-mm FWHM Gaussian filter before performing second-level random effects analyses across participants.

### Experimental design and statistical analysis

All statistical tests were performed across 42 participants (20 males, 22 females), with no between-subject factors. Behavioral analyses used repeated measures ANOVA to compare conditions. Univariate fMRI analyses used one-sample (paired-sample) two-tailed *t* tests to compare responses against baseline, between conditions, or linear contrasts of regression coefficients, and repeated measures ANOVA to compare multiple conditions. RSA fMRI analyses used one-sample one-tailed *t* tests to test for greater-than-chance representation of each information type, paired-sample two-tailed *t* tests to compare networks, and repeated measures ANOVA to test the interaction of information type and network. Within-subject factors are detailed in the relevant Results sections. For each analysis, multiple comparisons (across networks, component ROIs, or brain voxels) were accounted for by controlling the FDR at 0.05, unless noted otherwise. Effect sizes were calculated using partial η-squared for ANOVAs and Cohen's *d* for *t* tests. Analyses were performed using MATLAB (The MathWorks), SPM 12 (http://www.fil.ion.ucl.ac.uk/spm), and SPSS (version 25). In repeated measures ANOVA, Greenhouse–Geisser correction was used to adjust for non-sphericity. Data are available on request.

## Results

### Behavioral results

Overall accuracy was 97.5 ± 0.4% (mean ± SEM) and overall reaction time was 849 ± 23 ms. To match the fMRI analysis, the behavioral analysis discarded occasional episodes with >25% errors, where it was likely that the correct cue was not being followed (see Materials and Methods; zero to six discarded episodes across participants). For the remaining episodes, we calculated percentages of response errors, and mean reaction times on correct trials only.

**Figure 3.**
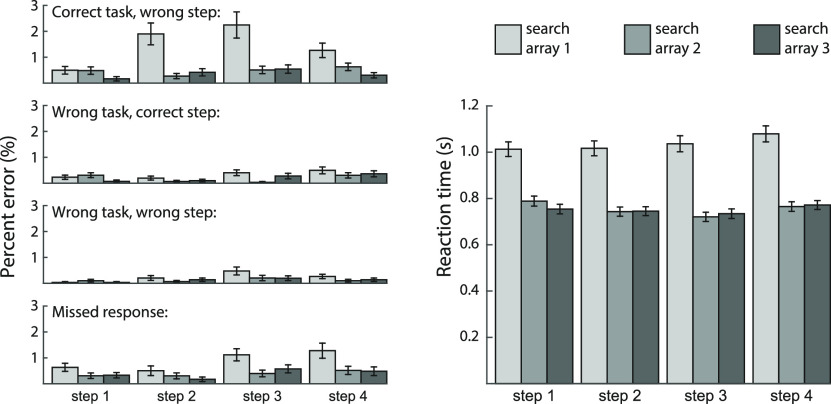
Behavioral performance summarized according to four possible error types (choosing an item from correct task but wrong step, wrong task but correct step, wrong task and step, and missed response), as well as reaction time for correct responses. For each step, the three bars indicate performance on each of the three successive search arrays. Error bars indicate SEM.

Error responses were broken into four error types (Fig. 3, left): choosing an item from the correct task but wrong step, wrong task but correct step, wrong task and step, and missed response. Results show poorest performance for the first search array of each step, when participants were required to switch from one step to the next. A step (steps 1–4) × search array (first, second, third within each step) × task ANOVA was performed for each type of error. All error types showed a main effect of step (all *F*_(3,123)_ > 3.35, all *p*s < 0.04, all η_p_^2^ > 0.08), and linear trend analyses indicated an overall increase in error across steps (all *F*_(1,41)_ > 4.25, all *p*s < 0.05, all η_p_^2^ > 0.09). A main effect of search array was found for all error types except for wrong task and step (all *F*_(2,82)_ > 3.72; *p*s < 0.04, η_p_^2^ > 0.08), reflecting higher errors on the first search array of each step. Finally, correct task wrong step errors showed a significant step × array interaction (*F*_(6,246)_ = 5.89, *p* < 0.001, η_p_^2^ = 0.16). There were no main effects of task, or interactions with task, for any error type (all *p*s > 0.08, all η_p_^2^ < 0.05).

A similar ANOVA for reaction time (Fig. 3, right) also showed a significant main effect for step (*F*_(3,123)_ = 15.14, *p* < 0.001, η_p_^2^ = 0.27), a significant main effect for search array (*F*_(2,82)_ = 215.42, *p* < 0.001, η_p_^2^ = 0.84), and a significant step × array interaction (*F*_(6,246)_ = 9.13, *p* < 0.001, η_p_^2^ = 0.18). In this analysis, there was also a significant main effect of task (*F*_(5,205)_ = 23.36, *p* < 0.001, η_p_^2^ = 0.36), as well as an interaction with step (*F*_(15,615)_ = 15.14, *p* < 0.001, η_p_^2^ = 0.27) but not with search array (*F*_(10,410)_ = 1.63, *p* = 0.13, η_p_^2^ = 0.04); the 3-way interaction was also significant (*F*_(30,1230)_ = 7.48, *p* < 0.001, η_p_^2^ = 0.15). RT varied idiosyncratically across tasks and steps, but in every case the first response was slowest. Across tasks and steps, the mean RT for the first response ranged from 0.88 to 1.14 s; for the second and third responses, mean RT ranged from 0.69 to 0.86 s.

### Univariate results

#### ROI analysis

**Figure 4.**
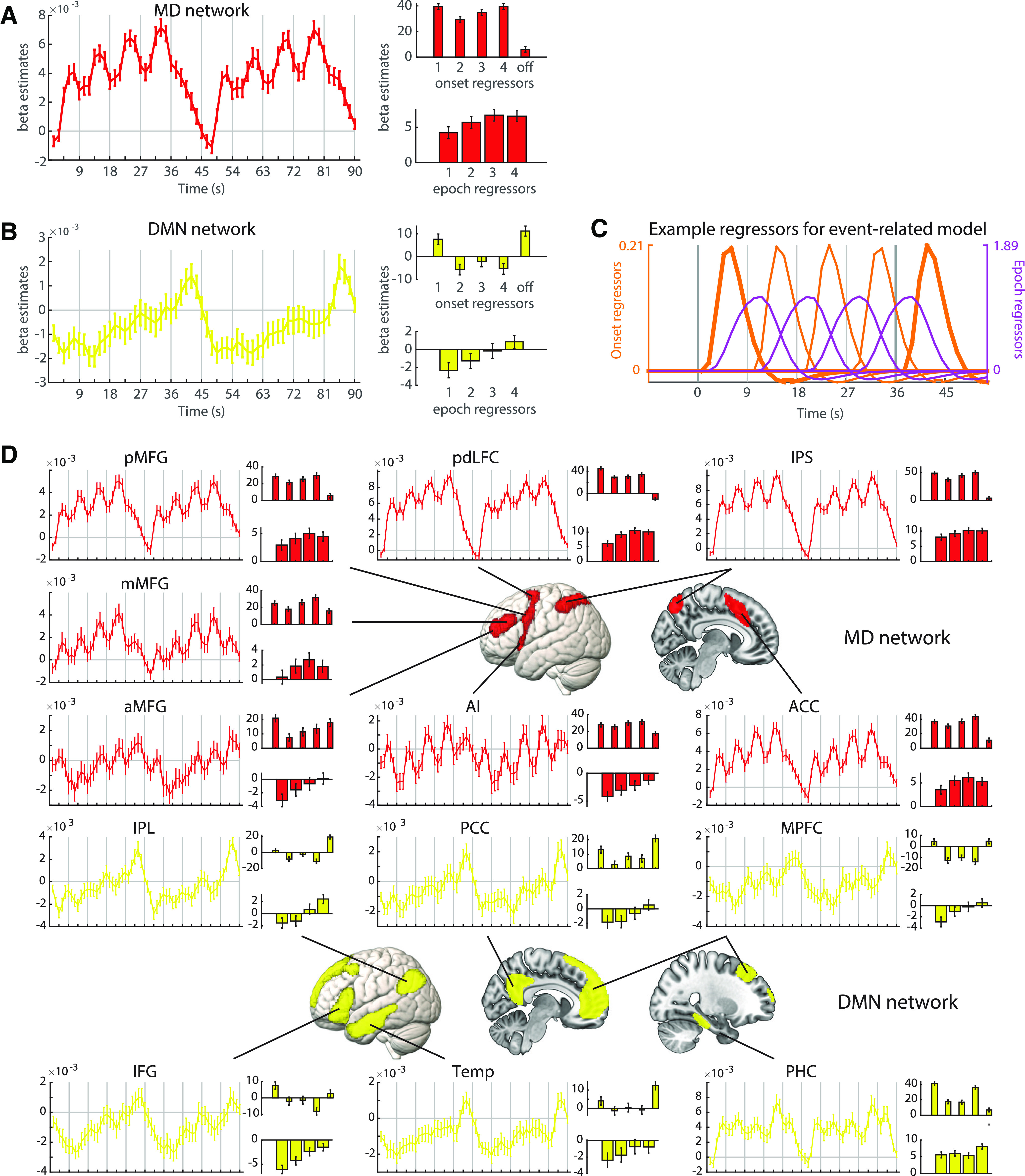
Univariate ROI analysis of MD network (***A***) and DMN (***B***) BOLD response across task episodes. For each network, the left plot shows results of the FIR analysis, with BOLD response as a function of time as participants progressed across two consecutive episodes. The upper right plot shows β estimates associated with step onset regressors (bars 1–4), and with the end of the episode (bar “off”). The lower right plot shows β estimates for epoch regressors per step. Error bars indicate SEM. ***C***, Example regressors modeling the transient onset/offset responses (orange) and the sustained epoch responses (purple) to each step within an episode. Vertical gray lines mark the beginning/end of each step. Thicker gray lines indicate episode boundaries; thicker orange lines indicate responses to these episode-boundary transitions. ***D***, FIR time-courses and activation profiles of onset and epoch responses in individual ROIs within the MD network (red) and DMN (yellow). The layout is the same as panel ***A***. aMFG/mMFG/pMFG: anterior, middle, and posterior middle frontal gyrus; pdLFC: posterior-dorsal lateral frontal cortex; IPS: intraparietal sulcus; AI: anterior insula; ACC: anterior cingulate cortex; IPL: inferior parietal lobule; PCC: posterior cingulate cortex; MPFC: medial prefrontal cortex; IFG: inferior frontal gyrus; Temp: lateral/anterior temporal cortex; PHC: parahippocampal cortex. For all panels, note that activation values associated with the FIR, onset, and epoch regressors are in arbitrary units as their scale depends on the regressor height.

The FIR model provided estimates of the observed BOLD response time course across a pair of task episodes, in successive 1.5 s windows starting from the onset of the first step. In the main analysis, we extracted these FIR responses from *a priori* networks (Fig. 4*A*,*B*, left). The MD network exhibited positive activity throughout each episode, along with four peaks corresponding to the four steps (Fig. 4*A*, left). These results suggest involvement in setting up and executing individual task steps. Additionally, overall MD activity gradually increased throughout the task episode, suggesting that the MD network is also sensitive to progress through the episode. For DMN regions, in contrast, tonic activation began below baseline, but also gradually increased through the episode, culminating in a large phasic response at episode completion (Fig. 4*B*, left). For both networks, the signal clearly resets between episodes.

To quantify the phasic and tonic components contributing to the BOLD response at each task step, we performed a complementary event-related GLM analysis with onset and epoch regressors modeling each task step (Fig. 4*A*,*B*, right). The regressors are illustrated in Figure 4*C* for a single episode. Four onset regressors were designed to reflect phasic activity at the onset of each task step. The final offset regressor was included to capture phasic activity at the end of the episode. Thus, the first onset regressor and the final offset regressor captured transient responses to episode boundaries, while the remaining onset regressors captured responses to step transitions within an episode. Finally, four epoch regressors were designed to reflect tonic activity throughout each step. Note that the activation values associated with the FIR, onset, and epoch regressors are in arbitrary units as their scale depends on the height of the regressors.

Within the MD network (Fig. 4*A*, right), there were strong onset responses, in line with FIR results. Contrasts with baseline showed that all four step onsets were significantly greater than baseline (all *t*s > 10.91, all *p*s < 0.001, all *d*s > 1.68) and there was a smaller yet significant offset response (*t* = 2.48, *p* = 0.02, *d* = 0.38). A one-way repeated measures ANOVA showed a significant difference across the four step onsets (*F*_(3,123)_ = 5.60, *p* < 0.01, η_p_^2^ = 0.12), with a quadratic (*F*_(1,41)_ = 21.61, *p* < 0.001, η_p_^2^ = 0.35) but not linear (*F*_(1,41)_ = 0.22, *p* = 0.64, η_p_^2^ < 0.01) trend across steps, reflecting an increasing response across steps 2–4, but a disproportionate response to the onset of the first step, i.e., the onset of the entire episode. Looking at epoch regressors, all four epoch responses were greater than baseline (all *t*s > 3.96, all *ps* < 0.001, all *d*s > 0.61). ANOVA showed a significant main effect of step (*F*_(3,123)_ = 7.73, *p* = 0.01, η_p_^2^ = 0.16), as well as a significant linear (*F*_(1,41)_ = 9.48, *p* < 0.01, η_p_^2^ = 0.19) and quadratic trend (*F*_(1,41)_ = 5.08, *p* = 0.03, η_p_^2^ = 0.11), reflecting an increasing but saturating response.

The DMN network showed a different profile (Fig. 4*B*, right). Only the onset of the first step (*t* = 3.22, *p* < 0.01, *d* = 0.50) and the offset response at the end of the episode (*t* = 4.38, *p* < 0.001, *d* = 0.68) were greater than baseline. Step onsets 2–4 were not significantly different from baseline (all |t|s < 2.09, all *p*s > 0.07, all |d|s < 0.33). ANOVA of the four step onsets showed a significant main effect of step (*F*_(3,123)_ = 9.87, *p* < 0.001, η_p_^2^ = 0.19), as well as significant linear (*F*_(1,41)_ = 9.70, *p* < 0.01, η_p_^2^ = 0.19) and quadratic (*F*_(1,41)_ = 7.16, *p* = 0.01, η_p_^2^ = 0.15) trends, consistent with the larger response to the first onset. Among the epoch responses, the first step was significantly lower than baseline (*t* = −3.21, *p* = 0.01, *d* = −0.49; for steps 2–4 all |t|s < 1.60, all *p*s > 0.23, all |d|s < 0.19). ANOVA showed a significant main effect of step (*F*_(3,123)_ = 18.42, *p* < 0.001, η_p_^2^ = 0.31), as well as a significant linear trend (*F*_(1,41)_ = 38.89, *p* < 0.001, η_p_^2^ = 0.49), suggesting an increase in activation across steps. As seen in the FIR time course, this implies a gradual release of tonic deactivation across the duration of the task episode.

To compare the response profile of the two networks directly, we performed a series of ANOVAs with network as an additional factor. A first ANOVA examined tonic responses, with factors of epoch response (steps 1–4) and network. There was a significant main effect of step (*F*_(3,123)_ = 15.25, *p* < 0.001) and network (*F*_(1,41)_ = 83.86, *p* < 0.001), but no interaction (*F*_(3,123)_ = 2.28, *p* = 0.08), suggesting the tonic increase was similar for both networks. A second ANOVA focused on sensitivity to episode boundaries, with factors of boundary response (step 1 onset, step 4 offset) and network. There were significant main effects for step (*F*_(1,41)_ = 29.84, *p* < 0.001) and network (*F*_(1,41)_ = 88.58, *p* < 0.001), and a significant interaction (*F*_(1,41)_ = 128.91, *p* < 0.001) that reflected strongest responses to episode onset in the MD network (Dosenbach et al., 2006), and strongest responses to episode offset in the DMN. A final ANOVA examined responses to step transitions within an episode, with factors of step onset (steps 2–4) and network. There were significant main effects of step (*F*_(2,82)_ = 4.23, *p* = 0.02) and network (*F*_(1,41)_ = 235.53, *p* < 0.001), as well as a significant interaction (*F*_(2,82)_ = 7.36, *p* = 0.001), reflecting stepwise increases in the MD network but not the DMN.

To examine whether the profiles of different regions within each network showed unique responses, we performed similar analyses on individual ROIs (Fig. 4*D*). Trends of activation across the four steps for individual ROIs were largely similar to the network in which they belong, although there were some differences between ROIs. Within the MD network, aMFG and AI showed negative epoch responses, in contrast to other regions. The episode offset response was also especially high in aMPFC and especially small in pdLFC and IPS. Within the DMN, PHC showed positive epoch responses, in contrast to other regions.

#### Whole-brain analysis

**Figure 5.**
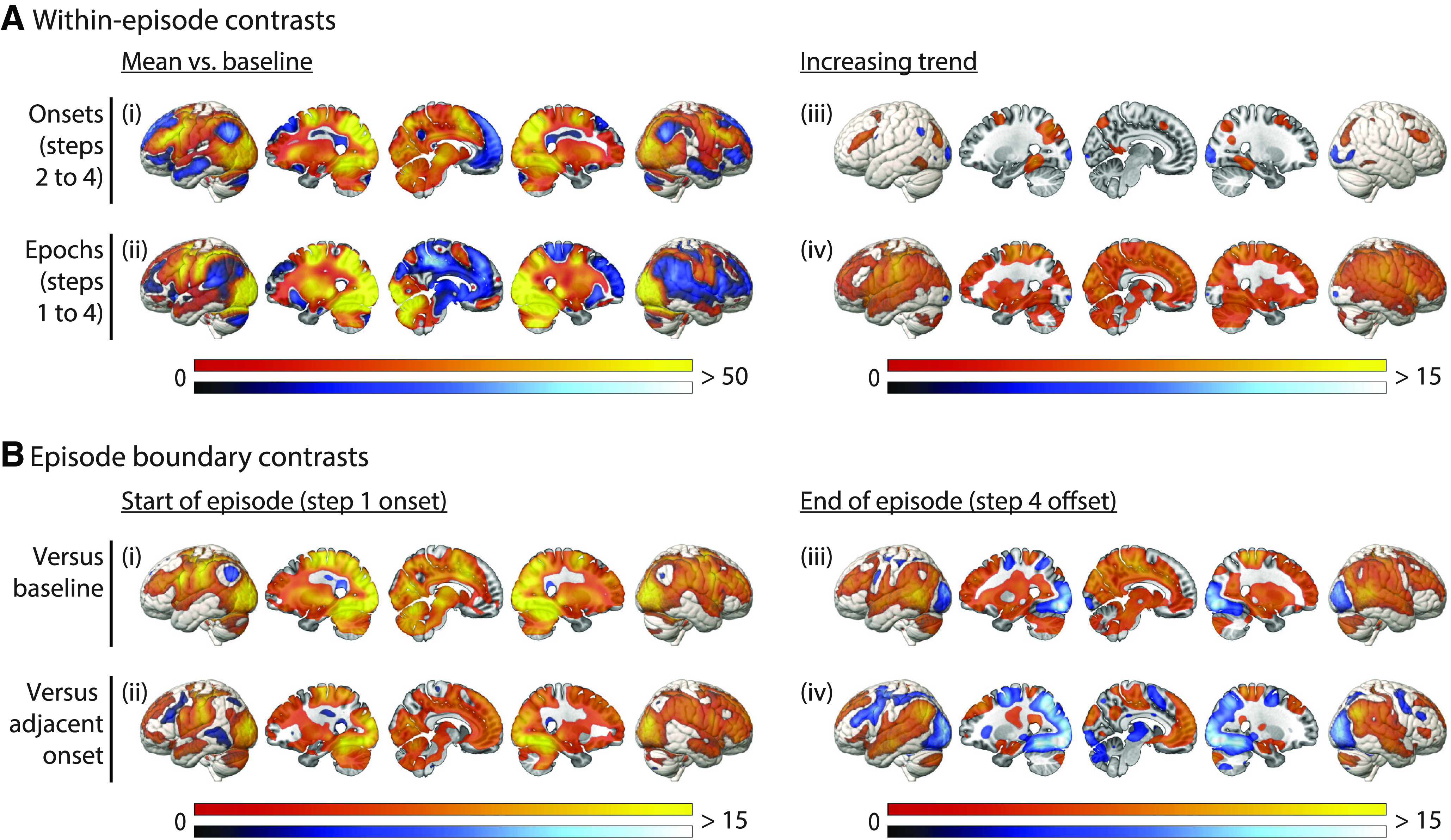
Whole-brain univariate analysis. ***A***, Responses within an episode, including (***i***) mean phasic responses to the onset of each step; (***ii***) mean tonic responses across the duration of each step; (***iii***) increases in the phasic response across step onsets; (***iv***) increases in the tonic response across step epochs. ***B***, Transient responses at episode boundaries, including (***i***) episode onset versus baseline; (***ii***) episode onset versus step 2 onset; (***iii***) episode offset versus baseline; (***iv***) episode offset versus step 4 onset. Colors indicate *t* values, with warm and cool scales indicating positive and negative tails, respectively. All activation maps are thresholded at FDR < 0.025 per tail.

Results from the whole-brain analysis, again separating onset and epoch regressors, are presented in Figure 5. Figure 5*A* shows responses within an episode, with the left-hand side showing contrasts of the mean onset (Fig. 5*Ai*) and epoch (Fig. 5*Aii*) response against baseline, and the right-hand side showing increasing trends across steps for the onset response (Fig. 5*Aiii*) and the epoch response (Fig. 5*Aiv*). The onset of step 1 and the offset of step 4 are special, since these correspond to the onset and offset of a whole episode, and it is evident from the ROI analysis that their neural response also differs from step onsets within an episode. Therefore, these episode-boundary responses were not included in the within-episode contrasts, but were instead examined separately. Figure 5*B* shows transient responses at episode onset (Fig. 5*B*, left) and offset (Fig. 5*B*, right), contrasted against both between-task baseline (Fig. 5*Bi*,*Biii*) and against the adjacent step onset response (Fig. 5*Bii*,*Biv*).

In comparison to baseline, the mean step onset response (Fig. 5*Ai*) was significantly positive throughout the MD network, as well as visual cortex, motor cortex, and subcortical structures including the cerebellum. The mean step onset response was significantly negative throughout the DMN. Mean epoch responses greater than baseline (Fig. 5*Aii*) were also extensive, including parietal and frontal regions overlapping with the MD ROIs, as well as expected regions of visual and motor cortex. Again, we saw negative epoch responses in much of the DMN. We next examined activity changes across steps within an episode. An increase in the amplitude of the step onset response was restricted to MD regions (Fig. 5*Aiii*). In contrast, a linear increase in the tonic epoch response was widespread across most of the brain (Fig. 5*Aiv*). The only exception was areas of visual cortex, where both onset and epoch responses decreased across an episode. Finally, we were interested in the response at episode boundaries, i.e., the onset of the first step (initiation of an episode) and the offset of the fourth step (completion of an episode). The response to step 1 onset was substantial across much of the brain, whether compared with baseline or to step 2 onset (Fig. 5*Bi*,*Bii*), including visual cortex and parts of DMN and MD networks. The episode completion response was also significantly greater than baseline in many brain regions (Fig. 5*Biii*), including parts of both MD and DMN networks, while deactivations were mainly observed in visual cortex. Interestingly, this response exceeded the previous (step 4) onset response in the DMN but not the MD network (Fig. 5*Biv*).

The results may be summarized as follows. Most MD regions, along with visual cortex, showed positive onset and epoch responses to all steps, suggesting direct involvement in setting up and executing task steps. DMN regions, in contrast, showed largely negative step and epoch responses. In much of the brain, sensitivity to the large-scale structure of the task episode was evident in gradually increasing activity as the episode progressed, along with phasic responses at onset and offset of the whole episode. Interestingly, increasing amplitude of phasic intra-episode step responses was highly specific to the MD network, and an episode offset response exceeding the preceding step onset was largely specific to the DMN.

### RSA results

**Figure 6.**
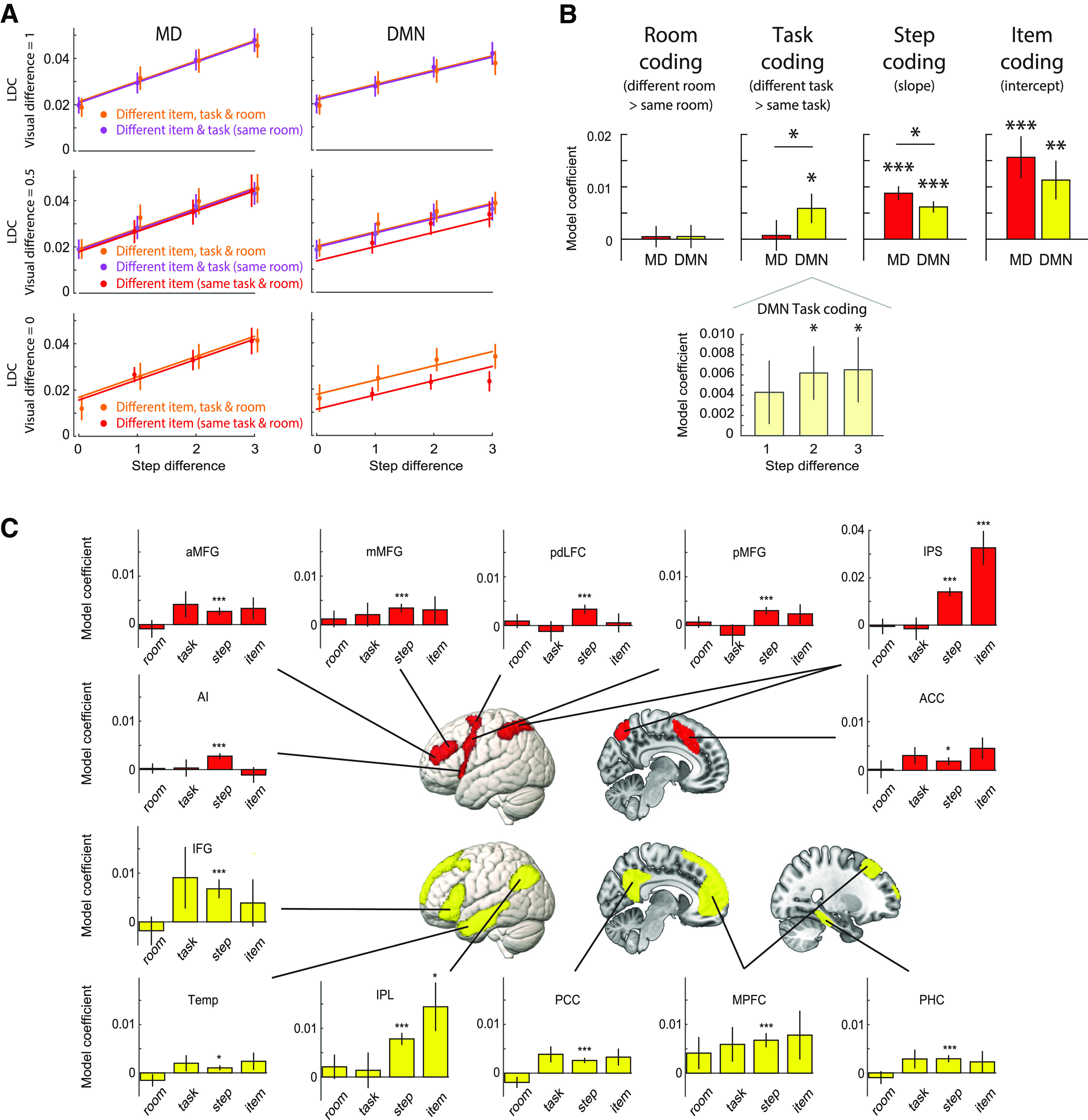
***A***, LDC representational distance estimates, modeled as in Figure 2*B*. Data points are plotted separately for different levels of visual difference (Fig. 2*C*) and are offset fractionally along the *x*-axis for visibility. Lines represent the mean linear model fit, estimated using all data (except matched-item comparisons). Visual difference = 0: displays based on items from same two tasks; difference = 0.5, one shared task; difference = 1, no shared tasks. Note that pairs from the same cued task (red) necessarily shared display items from at least one task (no data in top row), while different-task same-room pairs (purple) never shared items from two tasks (no data in bottom row). ***B***, top, LDC contrasts representing strength of room, cued task, step, and item representation in DMN and MD network-level ROIs. Asterisks above each bar indicate significance of one-tailed *t* tests against zero, after controlling FDR <0.05 across ROIs; horizontal lines indicate a significant two-tailed paired *t* test between networks. Bottom, Task representation broken down by step difference in the DMN network ROI. ***C***, Representation of room, task, step, and item information in individual ROIs in the MD network (red) and DMN (yellow). Asterisks indicate significance of 1-tailed t tests against zero, controlling FDR < 0.05 across ROIs, separately for each information type. All panels: error bars represent ±1 SEM across subjects; *p < 0.05, **p < 0.01, ***p < 0.001.

**Figure 7.**
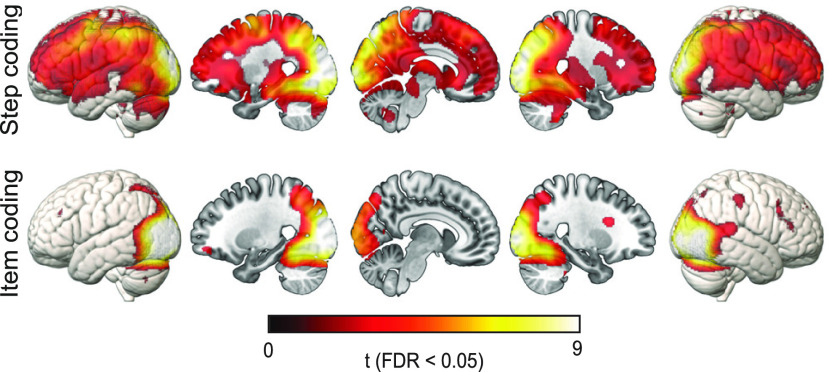
Representation of step and item across the whole brain, calculated using local spherical searchlights, and thresholded at FDR < 0.05. No voxels survived this threshold for room or task representation.

Results of the RSA analysis are shown in Figure 6. In Figure 6*A*, LDC representational distance estimates are plotted for various comparisons of events (see also Fig. 2), based on activation patterns across the DMN and MD networks. The coefficients of the linear model fit to these data are plotted in Figure 6*B*, quantifying representation of different types of information: room (greater LDC for different room than same room), cued task (greater LDC for different task then same task), step (the slope as a function of step difference), and item (measured by the intercept of the full model). Analyses of individual ROIs within the two networks are shown in Figure 6*C*. Results of a whole-brain searchlight analysis are shown in Figure 7.

#### Network comparison

First, we asked whether activity patterns in the MD and DMN networks differentially carried information about distinct aspects of task episodes. A 2 (network) × 4 (type of information) repeated measures ANOVA showed a significant interaction (*F*_(3,123)_ = 5.01, *p* = 0.006), as well as a main effect of information type (*F*_(3,123)_ = 4.90, *p* = 0.008) but not network (*F*_(1,41)_ = 0.36, *p* = 0.55). The interaction was driven by the DMN having a relative preference for representing the identity of the cued task, while the MD network had a relative preference for step-level representation (step position, and item identity). We next assessed representation of each information type in turn.

#### Room representation

Room representation would appear as a separation between the orange and purple lines in Figure 6*A*. Neither the DMN nor MD network ROIs showed significant room representation (both *t*s < 0.25, both *p*s > 0.39, both *d*s < 0.04), and there was no difference between the networks (*t*_(41)_ = 0.023, *p* = 0.98). Similarly, none of the individual ROIs showed significant room representation (all *t*s < 1.70, all *p*s > 0.11, *d*s < 0.27), although it was numerically strongest in the MPFC. No voxels survived FDR correction in the whole-brain searchlight analysis.

#### Task representation

Representation of the cued task appears as a separation between the red and purple lines in the middle row of Figure 6*A*. Given no effect of room, converging evidence comes from the separation of red and orange lines in the bottom row. The DMN network ROI showed significant representation of the cued task (*t*_(41)_ = 2.18, *p* = 0.02, *d* = 0.33), while the MD network ROI did not (*t*_(41)_ = 0.26, *p* = 0.40, *d* = 0.04); the difference between networks was also significant (*t*_(41)_ = 2.52, *p* = 0.02, *d* = 0.39). None of the individual ROIs showed significant task representation after FDR correction for multiple comparisons across ROIs. PCC and ACC showed task representation before correction (both *t*s > 1.74, both *p*s < 0.044, both *d*s > 0.27). Task representation was positive in all six DMN ROIs, but only four of seven MD ROIs. No voxels survived FDR correction in the whole-brain searchlight analysis.

It is possible that the response to regressors modeling adjacent steps could be similar because of imperfect temporal separation of the signal, such that pairs of steps within the same task appear more similar than those from different tasks because of differences in temporal separation in addition to differences in task identity. We examined this possibility by fitting four separate linear regression models using subsets of cells, chosen to differ in separation of one, two, or three steps. That is, we extracted LDC values from cells of the DMN network RDM that represented one step apart (1 vs 2, 2 vs 3, and 3 vs 4), two steps apart (1 vs 3 and 2 vs 4), or three steps apart (1 vs 4), and, in each case, fitted a model with room, cued task, and visual difference regressors. If temporal proximity were contributing to activity pattern similarity, and hence to apparent task representation in the DMN, we should expect a stronger effect for steps closer together in time. However, we found no evidence of any difference in task representation across these three conditions (*F*_(2,82)_ = 0.39, *p* = 0.61, η_p_^2^ = 0.01), nor a linear trend as a function of step (*F*_(1,41)_ = 0.44, *p* = 0.51, η_p_^2^ = 0.01). Task representation within the DMN is shown broken down by step difference in Figure 6*B*.

#### Step representation

Step representation, visible as the linear slopes in Figure 6*A*, was significant in both the DMN (*t*_(41)_ = 6.34, *p* < 0.001, *d* = 0.98) and MD (*t*_(41)_ = 7.25, *p* < 0.001, *d* = 1.12) network ROIs. The MD network showed significantly greater step representation than the DMN (*t*_(41)_ = 2.38, *p* = 0.02, *d* = 0.37). Step representation was also significant in all the individual ROIs (all *t*s > 2.02, all *p*s < 0.03, all *d*s > 0.31). This was not surprising, as in our univariate analysis we observed strong linear trends across the episode for most of the brain (Fig. 5*Aiv*). Similarly, in the whole-brain searchlight analysis (Fig. 7), step representation was significant across most of the brain, although strongest in visual cortex and with local peaks in MD regions.

#### Item representation

Both DMN (*t*_(41)_ = 3.15, *p* = 0.002, *d* = 0.49) and MD (*t*_(41)_ = 4.00, *p* < 0.001, *d* = 0.62) networks showed significant representation of item, visible as a positive intercept in the lower row of Figure 6*A*. The two networks did not significantly differ in item representation (*t*_(41)_ = 1.88, *p* = 0.07, *d* = 0.29). In the individual ROIs, item representation was especially strong in parietal regions, with only IPS (*t*_(41)_ = 4.58, *p* < 0.001, *d* = 0.71) and IPL (*t*_(41)_ = 2.89, *p* < 0.01, *d* = 0.44) showing significant item representation after FDR correction for multiple comparisons [before correction, item representation was also present in ACC (*t*_(41)_ = 2.05, *p* = 0.02, *d* = 0.32) and PCC (*t*_(41)_ = 1.90, *p* = 0.03, *d* = 0.29)]. Item representation was positive in all six DMN ROIs and six of seven MD ROIs. In the whole-brain searchlight analysis (Fig. 7), item representation was strongest in visual cortex, extending into the parietal lobe, especially along the IPS, and with scattered foci in lateral frontal regions.

#### Results are not explained by differences in reaction time

Since RTs were faster for some tasks and items than others, RT differences could conceivably contribute to neural pattern differences between conditions. To test this, we performed a control analysis that added cross-validated RT difference as a covariate when modeling neural pattern difference between conditions. RT difference did not explain unique variance in neural pattern difference for either network (MD: *t*_(41)_ = −1.04, *p* = 0.85, *d* = −0.16; DMN: *t*_(41)_ = −1.39; *p* = 0.91, *d* = −0.21), and, importantly, its inclusion in the model did not change the main findings. Specifically, the interaction between network and type of represented information remained (*F*_(3,123)_ = 3.75, *p* = 0.02, η_p_^2^ = 0.08); task was represented in the DMN (*t*_(41)_ = 2.08, *p* = 0.02, *d* = 0.32) but not the MD network (*t*_(41)_ = 0.26, *p* = 0.40, *d* = 0.04), with a significant difference between networks (*t*_(41)_ = 2.29, *p* = 0.03, *d* = 0.35), and the DMN task representation not differing with step distance (*F*_(2,82)_ = 0.59, *p* = 0.53, η_p_^2^ = 0.01); step was represented in the DMN (*t*_(41)_ = 7.32, *p* < 0.001, *d* = 1.13) and the MD network (*t*_(41)_ = 8.03, *p* < 0.001, *d* = 1.24), but more strongly in the MD network (*t*_(41)_ = 2.84, *p* = 0.007, *d* = 0.44); item was represented in the DMN (*t*_(41)_ = 2.88, *p* = 0.003, *d* = 0.44) and the MD network (*t*_(41)_ = 3.39, *p* = 0.001, *d* = 0.52), with no difference between them (*t*_(41)_ = 1.26, *p* = 0.22, *d* = 0.19). Thus, we find no evidence that the difficulty of particular conditions, as indexed by RT, explains the observed pattern differences, or representation of step, item, or task.

To summarize, we found that both networks represent the content (item) and sequential position (step) of individual subgoals, but the MD network favors this step-level information, while the hierarchically higher level of task identity is preferentially represented in the DMN. We found no reliable evidence for representation of task groupings (room) at the highest hierarchical level.

## Discussion

The DMN and MD networks are expected to jointly support memory-guided cognitive control (Margulies and Smallwood, 2017). Using fMRI, we examined how they respond to and represent different aspects of multistep task episodes. MD regions responded positively throughout an episode, with separate peaks for successive steps. The DMN instead showed overall deactivation, and minimal response to intra-episode step transitions. Both networks, with widespread other regions, were sensitive to large-scale episode structure, exhibiting phasic responses to episode onset, and gradually increasing tonic activity across an episode. MD regions uniquely showed progressively increasing phasic step responses, while an episode offset response exceeding the final step response was characteristic of the DMN. RSA revealed distinct information profiles within the networks. The MD system represented individual items but not cued tasks, while the DMN represented both items and tasks. Step was represented by both networks, but more strongly by the MD network.

We consider a temporal hierarchy of task goals, with lower-level items/steps nested within higher-level tasks/episodes. Thus, multivariate item representation, plus phasic univariate responses per step, imply sensitivity to the lower level; task representation, plus univariate responses to episode boundaries and intra-episode trends, indicate sensitivity to the higher level. Step representation is more ambiguous, implying multilevel information by indexing a step's position within an episode. Thus, we do not find an exclusive mapping between networks and levels of the task hierarchy; rather, both networks are sensitive to both levels. Similarly, both networks exhibit slow dynamics (ramping epoch responses) and fast dynamics (transient responses to episode boundaries, plus steps in MD regions). Nonetheless, whenever networks differed, in either univariate response or multivariate representation, it suggested preferential step-level and episode-level sensitivity in MD and DMN regions, respectively. This is consistent with closer coupling of MD regions to moment-by-moment perception and action, while the DMN is maximally distant from sensorimotor cortex (Margulies et al., 2016). Although we do not attempt to map the task hierarchy onto a neural hierarchy, such relationships may exist both at scales more global (Vidaurre et al., 2017) and more local (Badre and Nee, 2018) than the networks considered here.

Relative sensitivity of MD regions to step transitions, identity, and item content, aligns with prior research. Many experiments demonstrate representation of task-relevant items in MD regions (Freedman et al., 2001; Li et al., 2007; Woolgar et al., 2011), with radical reorganization between task steps (Sigala et al., 2008), and MD activity at transitions between events and subgoals (Sridharan et al., 2007; Farooqui et al., 2012). Together with these previous findings, our results suggest that, as a task episode progresses, MD representations in particular are in constant flux, reorganizing to represent the detailed contents of each step. Representational content includes the step's position within the episode and the identity of the associated item, which in our task may serve as an attentional template for visual search decisions (Desimone and Duncan, 1995), consistent with strong item representation also in occipital regions.

In contrast, the DMN responded strongly at episode boundaries, without significant responses to intermediate step transitions. This echoes reports of DMN activation at boundaries between extended events (Speer et al., 2007), and at transitions to new tasks (Smith et al., 2018). Our data showed, however, that episode onset and offset responses were both widespread in the brain (Fox et al., 2005a), while the relative magnitude of the offset response was most specific to the DMN. Possibly, the DMN, along with other brain regions, is involved in long-term memory retrieval of an entire task sequence at episode initiation, and consolidation at episode completion (Schneider and Logan, 2006; Farooqui and Manly, 2019). Whether these findings depend on sematic knowledge associated with our life-like tasks (Humphreys et al., 2015; Murphy et al., 2018) requires further experimentation. Marked DMN responses to episode boundaries but not step transitions, along with representation of task identity, support proposals that the DMN represents information that remains stable over long timescales (Lerner et al., 2011; Chen et al., 2016).

The DMN also represented items, i.e., specific elements within an episode as well as broader task context. Joint representation of both hierarchical levels aligns with the concept of a “situation model,” a cognitive representation of relationships between elements of an episode (Ranganath and Ritchey, 2012). More anterior DMN subregions are implicated in schema representation, capturing similarities across multiple episodes (Preston and Eichenbaum, 2013; Ghosh and Gilboa, 2014; Robin and Moscovitch, 2017), so might have been expected to represent task groupings by room. Room representation was numerically strongest in the MPFC, but not significant. Stronger room representation might require grouping of tasks to be behaviorally relevant rather than incidental. Despite item and task representations coexisting in the DMN, consistent with a compositional code, this experiment cannot determine whether they are bound into conjunctive representations, or maintained as independent factorized components (Behrens et al., 2018): because items were task-unique, item-task conjunctions are indistinguishable from item representation. Disentangling these different forms of co-representation requires the same item to appear in different contexts. Such designs have identified item-context conjunctions in the hippocampus (Hsieh et al., 2014), item-order associations in frontal and temporal regions (Reverberi et al., 2012; Kalm and Norris, 2014), rule-rule compositionality in lateral frontal cortex (Cole et al., 2011), and factorized sequence and position codes in mid-cingulate cortex (Holroyd et al., 2018) and in electrophysiological signals during learning and replay (Liu et al., 2019).

Both networks, along with most brain regions, tracked intra-episode progress, shown by ramping univariate responses. This is consistent with reports of increasing activity across task episodes in specific MD (Farooqui et al., 2012; Desrochers et al., 2015, 2019) and DMN regions (Vatansever et al., 2017) but suggests a very global property of brain function (Farooqui and Manly, 2018). While some visual areas showed decreasing activity, perhaps reflecting adaptation to sensory input (Grill-Spector et al., 2006), most regions showed gradually increasing activity, which reset between episodes. As this effect was so widespread, it is difficult to offer a precise interpretation, and different areas may increase for different reasons (Kalm and Norris, 2017). For example, ramping responses could variously reflect monitoring or reconfiguration of control representations that may increase in demand as an episode unfolds (Farooqui et al., 2012; Desrochers et al., 2015, 2019); a transition from effortful rule retrieval to more automatic responding (Vatansever et al., 2017); reducing prospective memory load (Momennejad and Haynes, 2012) as steps are completed; increasing expectation of episode completion (Shidara and Richmond, 2002); or integration of information into an episode representation (Hasson et al., 2008; Dumontheil et al., 2011; Lerner et al., 2011). A global ramping response is also reminiscent of models of multistep decision-making, in which evidence accumulation is massively parallel, within serially-chained temporal chunks (Zylberberg et al., 2011; Dehaene and Sigman, 2012). In rats, anticipation of distant goals has been associated with slowly ramping dopamine release (Howe et al., 2013), suggesting a potential mechanism for such widespread cortical effects. Contrasting with the global nature of the tonically increasing response, progressive increases in the phasic step response appeared highly specific to MD regions. Speculatively, phasic MD responses may track progress in discrete steps, whereas global ramping signals reflect a more continuous measure of progress. A similar distinction between neural signals that track progress in smooth versus action-linked manners has also been observed in the rat (Ma et al., 2014).

Since opposing sensitivity to task difficulty is characteristic of both networks, cognitive demand could potentially influence the current findings. Behavioral results confirm that difficulty varies across an episode. Multiple cognitive factors undoubtedly contribute, as discussed above, requiring additional experiments to distinguish. However, while some univariate findings match classical observations of opposing “task-negative” versus “task-positive” DMN and MD network responses, respectively (e.g., mean activation/deactivation during the task, vs intertrial baseline) other results are not easily explained in this way. One example is the tonically increasing response, which follows the same trend for both networks. Regarding RSA, modeling cross-validated between-condition RT differences provided no evidence that difficulty, as indexed by RT, explained unique variance in pattern differences, or contributed to step, item, or task representation. We also note that a simple difficulty-based effect would not obviously explain the crossed interaction between network and represented information type.

Hierarchical control structures link task goals, context, specific actions, and serial position codes, allowing learned rules to guide ongoing behavior (Rosenbaum et al., 1983; Schneider and Logan, 2006; Badre, 2008). Our results describe how broad brain networks collaborate in episodic control of task sequences, with MD and DMN regions exhibiting distinct time-courses throughout the episode, and different profiles of information representation. The DMN may link individual cognitive operations and their broader context, consistent with a “situation model” (Ranganath and Ritchey, 2012). The MD system, along with sensory regions, tracks the detailed content of individual cognitive operations, locked to discrete events within the episode. Both networks respond to the broad temporal structure of task episodes, with phasic activity at episode boundaries, and gradually increasing activity within an episode. Acting together, they reflect the hierarchical structure of goal-directed behavior.
